# The TSPO Ligands 2-Cl-MGV-1, MGV-1, and PK11195 Differentially Suppress the Inflammatory Response of BV-2 Microglial Cell to LPS

**DOI:** 10.3390/ijms20030594

**Published:** 2019-01-30

**Authors:** Maya Azrad, Nidal Zeineh, Abraham Weizman, Leo Veenman, Moshe Gavish

**Affiliations:** 1 The Ruth and Bruce Rappaport Faculty of Medicine, Technion Institute of Technology, Haifa 31096, Israel; mayabz@gmail.com (M.A.); nidalz1988@gmail.com (N.Z.); jehudaveenman@gmail.com (L.V.); 2 Research Unit at Geha Mental Health Center and the Laboratory of Biological Psychiatry, Felsenstein Medical Research Center, Petah Tikva 4910002, Israel; weizmana@gmail.com; 3 Sackler Faculty of Medicine, Tel Aviv University, Tel Aviv 6997801, Israel

**Keywords:** TSPO ligands, neuroinflammation, microglial activation, lipopolysaccharide (LPS), cyclooxygenase-2 (COX-2), inducible nitric oxide synthase (iNOS), nitric oxide (NO)

## Abstract

The 18 kDa Translocator Protein (TSPO) is a marker for microglial activation as its expression is enhanced in activated microglia during neuroinflammation. TSPO ligands can attenuate neuroinflammation and neurotoxicity. In the present study, we examined the efficacy of new TSPO ligands designed by our laboratory, MGV-1 and 2-Cl-MGV-1, in mitigating an in vitro neuroinflammatory process compared to the classic TSPO ligand, PK 11195. We exposed BV-2 microglial cells to lipopolysaccharide (LPS) for 24 h to induce inflammatory response and added the three TSPO ligands: (1) one hour before LPS treatment (pretreatment), (2) simultaneously with LPS (cotreatment), and (3) one hour after LPS exposure (post-treatment). We evaluated the capability of TSPO ligands to reduce the levels of three glial inflammatory markers: cyclooxygenase-2 (COX-2), inducible nitric oxide synthase (iNOS), and nitric oxide (NO). We compared the effects of the two novel ligands to PK 11195. Both 2-Cl-MGV-1 and MGV-1 reduced the levels of glial COX-2, iNOS, and NO in LPS-treated BV-2 cells more efficiently than PK 11195. Notably, even when added after exposure to LPS, all ligands were able to suppress the inflammatory response. Due to their pronounced anti-inflammatory activity, 2-Cl-MGV-1 and MGV-1 may serve as potential therapeutics in neuroinflammatory and neurodegenerative diseases.

## 1. Introduction

Neuroinflammation is implicated in the pathophysiology of various brain diseases and brain injuries, and is associated with a broad range of neuroimmune responses. Such responses typically involve the blood–brain barrier (BBB), glial cells, and neurons [1]. Microglial cells constitute a major component of the neuroinflammatory process and are the primary innate immune cells of the central nervous system (CNS) [2]. Upon stimulus, microglial cells switch to their activated state and function as phagocytes. Activated microglia release proinflammatory mediators, including induced nitric oxide synthase (iNOS), nicotinamide adenine dinucleotide phosphate (NADPH) oxidase, and cyclooxygenase 2 (COX-2), as well as cytokines (e.g., IL-1β, IL-6, and TNF-α), chemokines (e.g., MCP-1), nitric oxide (NO), and prostaglandins (PGs) [3].

The microglial response restricts the first stimuli and maintains tissue homeostasis [1]. However, exaggerated response is often accompanied by disruption of the BBB and neuronal damage due to the microglial neurotoxic activity [2]. Consequently, neuroinflammation may result in various adverse outcomes including synaptic dysfunction [4], inhibition of neurogenesis [5], and neuronal death [6,7]. Thus, it is not surprising that chronic neuroinflammation and microglial activation are associated with various neurodegenerative diseases, such as encephalitis [8], Alzheimer’s disease [9,10,11], Parkinson’s disease [12], multiple sclerosis (MS) [13], and amyotrophic lateral sclerosis (ALS) [14].

One characteristic feature of activated microglia following CNS injury is an increased expression of the translocator protein (TSPO), a mitochondrial protein that is involved in various cellular functions such as programmed cell death [15,16,17], regulation of cell nuclear gene expression [18,19], cell proliferation [20,21], cell differentiation [22], cholesterol transport [23,24], and regulation of steroidogenesis [25,26]. Several studies have shown increased binding of TSPO ligands in activated microglia as a result of CNS injury [27,28,29,30]. TSPO has become a marker for neuroinflammation, and its ligands are now used to image activated microglia in vivo [31]. Moreover, increased TSPO expression is seen in various neurodegenerative diseases that involve neuroinflammation [23,26,32,33,34,35,36,37,38,39,40].

Alterations of TSPO function modulate neuroinflammation and neurotoxicity [41,42,43,44]; for example, TSPO ligands treatment or TSPO overexpression in LPS-treated microglial cells reduces proinflammatory cytokine release whereas TSPO knockdown increases cytokine production [41]. Furthermore, TSPO ligands were shown to exert anti-inflammatory effects in vivo. One study has shown that PK 11195 and three other TSPO ligands diminished microglial activation and promoted neuronal survival in quinolinic acid-injected rats, which represents a model for excitotoxic neurodegeneration [43].

In the present study, we examined the efficacy of two new TSPO ligands, 2-(2-chlorophenyl) quinazolin-4-yl dimethylcarbamate (2-Cl-MGV-1) and 2-phenylquinazolin-4-yl dimethylcarbamate (MGV-1) [22], designed by our laboratory, in diminishing neuroinflammation in comparison to the classic TSPO ligand, PK 11195. Specifically, we evaluated the capability of 2-Cl-MGV-1 and MGV-1 to reduce the levels of inflammatory markers, including COX-2, iNOS, and NO, in LPS-induced microglial cells.

2-Cl-MGV-1 and MGV-1, among several others, are tricyclic compounds that were developed by expanding on a quinazoline scaffold [19]. Elongation of the alkyl side chains improved the affinity to TSPO. Although 2-Cl-MGV-1 and MGV-1 show lower affinities to TSPO, compared to other newly developed ligands and the classical TSPO ligand PK 11195, they have the advantage of showing very few lethal effects, even at high concentrations (100 µM), while still protecting against neuronal damage [22]. Classical TSPO ligands typically show pronounced lethal effects at high concentrations [17,45].

## 2. Results

### 2.1. The Chemical Structure and Characteristics of the New TSPO Ligands

The chemical structure of 2-Cl-MGV-1 and MGV-1 were described previously [22].

2-Cl-MGV-1 is 2-(2-chlorophenyl) quinazolin-4-yl dimethylcarbamate and MGV-1 is 2-phenylquinazolin-4-yl dimethylcarbamate. The affinity of both 2-Cl-MGV-1 and MGV-1 towards TSPO is 825 nM as previously described [22]. No toxic effects were detected in the liver and in the kidney of mice following two-month treatment with the two ligands in a dosage of 15 mg/kg. 

### 2.2. Effect of TSPO Ligands on COX-2 Expression Levels in LPS-Treated BV-2 Cells

#### 2.2.1. Pretreatment

**Figure 1:**
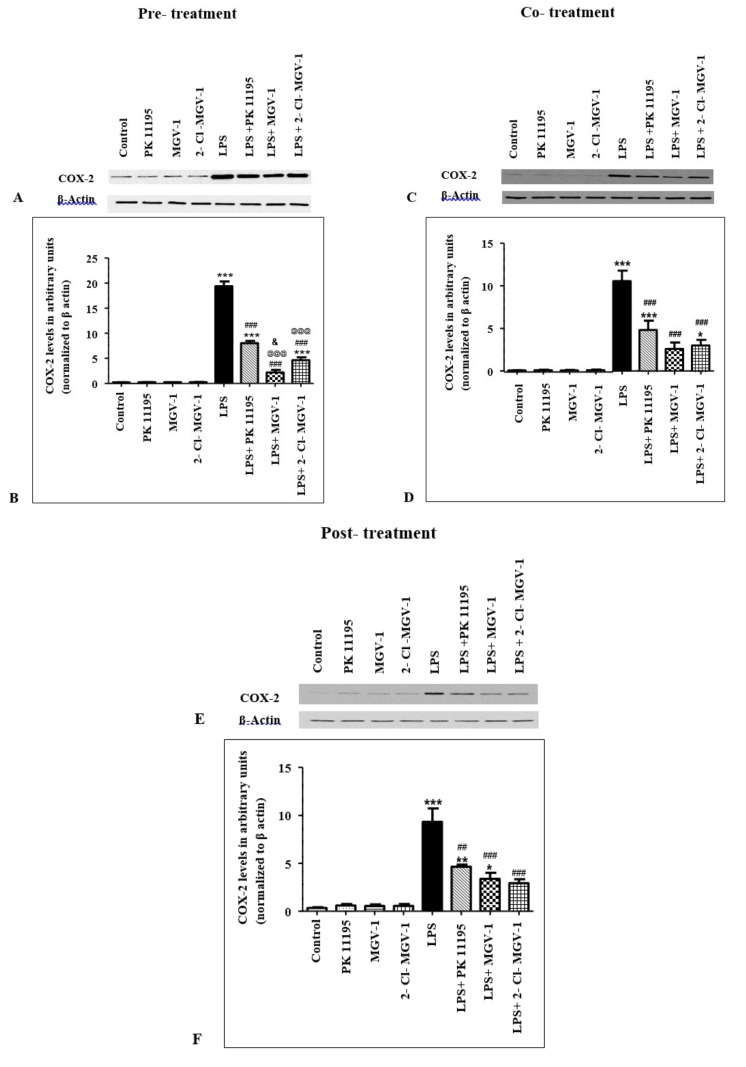
TSPO ligands reduced COX-2 expression levels in LPS-treated BV-2 cells. BV-2 cells were treated with 100 ng/mL LPS for 24 h, with or without TSPO ligands (25 μM) in three different scenarios. **A**,**C,E**: Representative Western blots of COX-2 and β-actin expression levels in pretreatment, cotreatment, and post-treatment, respectively, with the three TSPO ligands. **B,D,F**: Relative COX-2 protein expression levels normalized to β-actin in pretreatment, cotreatment, and post-treatment, respectively, with TSPO ligands. Results were calculated using densitometry and presented as means ± SEM; *n* = 4 (pretreatment), *n* = 5 (cotreatment), *n* = 3 (post-treatment). * *p* < 0.05, ** *p* < 0.01, *** *p* < 0.001 compared to Control; ## *p* < 0.01, ### *p* < 0.001 compared to LPS; @@@ *p* < 0.001 compared to LPS + PK 11195; & *p* < 0.05 compared to LPS + MGV-1.

Pretreatment with 25 µM PK 11195, 2-Cl-MGV-1, and MGV-1 in the absence of LPS did not affect COX-2 levels in BV-2 cells (ANOVA *p* < 0.0001) (Figure 1A,B). LPS treatment increased COX-2 levels in BV-2 cells 91-fold, compared to control untreated BV-2 cells (Bonferroni *p* < 0.001). Pretreatment with PK 11195 one hour prior to LPS treatment resulted in reduction of COX-2 levels by 59% (Bonferroni *p* < 0.001), while pretreatment with 2-Cl-MGV-1 and MGV-1 reduced COX-2 levels by 89% and 76%, respectively, compared to LPS-treated cells (Bonferroni *p* < 0.001 for both). Post hoc analysis showed that the effects of pretreatment with 2-Cl-MGV-1 or MGV-1 on COX-2 levels were significantly greater than the effect of pretreatment with PK 11195 (Bonferroni *p* < 0.001, for each comparison).

COX-2 levels in LPS + 2-Cl-MGV-1-treated BV-2 cells were significantly lower than COX-2 levels in LPS + MGV-1-treated BV-2 cells (Bonferroni *p* < 0.05). Moreover, comparison of COX-2 levels in LPS + ligand-treated cells to control untreated cells showed that COX-2 levels only in LPS + 2-Cl-MGV-1 did not differ significantly from COX-2 levels in control untreated cells (Bonferroni *p* > 0.05 for LPS + 2-Cl-MGV-1 vs. Control, *p* < 0.001 for LPS + PK 11195 vs. Control, *p* < 0.001 for LPS + MGV-1 vs. Control).

#### 2.2.2. Cotreatment

Similar to the results of the pretreatment scenario, the COX-2 levels in BV-2 cells treated with 25 µM of the TSPO ligands, in the absence of LPS, did not differ from COX-2 levels in untreated control cells (Figure 1C,D, ANOVA *p* < 0.0001). COX-2 levels were elevated following exposure to LPS, while cotreatment of LPS with each of the three tested TSPO ligands reduced COX-2 expression levels compared to the group of LPS-treated cells. Notably, while LPS + PK 11195 cotreatment reduced COX-2 levels by 54% (Bonferroni *p* < 0.001), LPS + 2-Cl-MGV-1 and LPS + MGV-1 cotreatment decreased COX-2 levels by 75% and 71%, respectively, compared to LPS treatment alone (Bonferroni *p* < 0.001, for each comparison). Interestingly, among the three ligands, cotreatment of LPS with either 2-Cl-MGV-1 or MGV-1 managed to decrease COX-2 to a level that was not significantly different from COX-2 protein levels in untreated control cells (Bonferroni *p* > 0.05 for LPS + 2-Cl-MGV-1 vs. Control, *p* > 0.05 for LPS + MGV-1 vs. Control, *p* < 0.001 for LPS + PK 11195 vs. Control).

#### 2.2.3. Post-Treatment

Subsequently, we evaluated the ability of 2-Cl-MGV-1, MGV-1, and PK11195 to attenuate the inflammatory response one hour after it has begun. Namely, BV-2 cells were treated with TSPO ligands (25 µM) one hour after the addition of LPS (the third scenario: post-treatment). Again, as with the former two scenarios, TSPO ligands in the absence of LPS did not influence COX-2 levels (Figure 1E,F, ANOVA *p* < 0.0001). Post-treatment with PK 11195, 2-Cl-MGV-1, and MGV-1decreased COX-2 levels by 50% (Bonferroni *p* < 0.001), 64% (Bonferroni *p* < 0.001), and 69% (Bonferroni *p* < 0.001), respectively, compared to LPS-treated BV-2 cells. COX-2 levels in LPS + MGV-1-treated cells did not differ significantly from COX-2 levels in control untreated BV-2 cells (Bonferroni *p* > 0.05). In contrast, after treatment with PK 11195 or 2-Cl-MGV-1, COX-2 levels in LPS-treated cells remained still significantly higher than in untreated control cells (*p* < 0.01 for LPS + PK 11195 vs. Control, *p* < 0.05 for LPS + 2-Cl-MGV-1 vs. Control).

### 2.3. Effect of TSPO Ligands on iNOS Expression Levels in LPS-Treated BV-2 Cells

#### 2.3.1. Pretreatment

**Figure 2:**
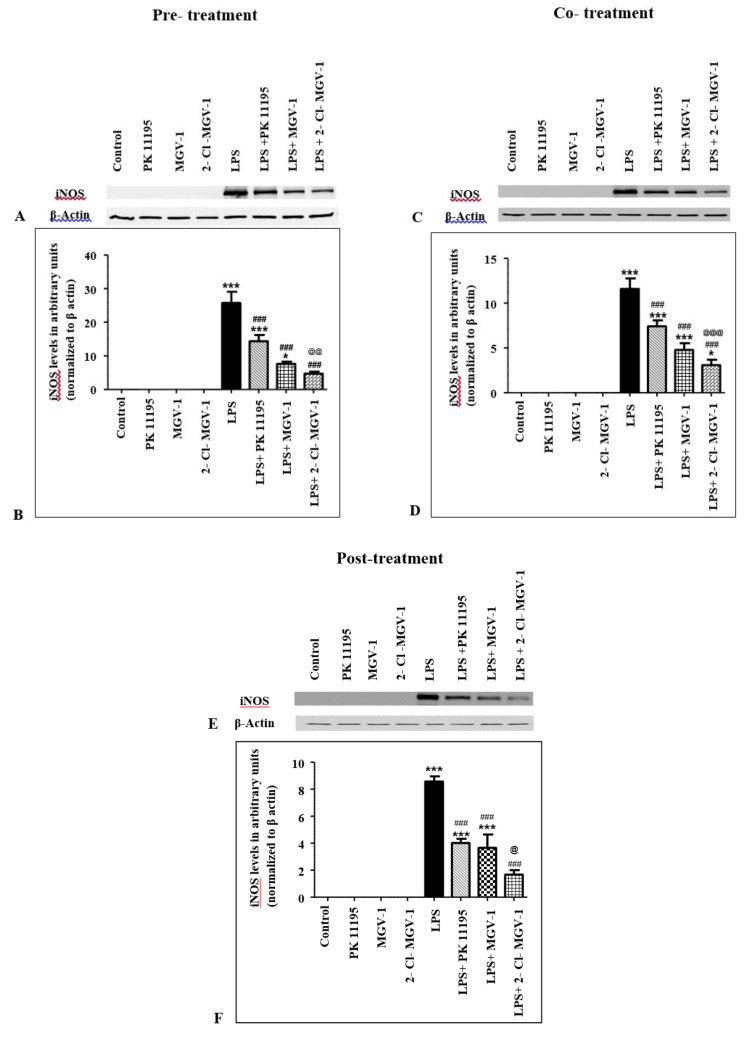
TSPO ligands reduced iNOS expression levels in LPS-treated BV-2 cells. BV-2 cells were treated with 100 ng/mL LPS for 24 h, with or without TSPO ligands (25 μM) in three different scenarios. **A**,**C,E**: Representative Western blots of iNOS and β-actin expression levels in pre-treatment, co-treatment, and post-treatment, respectively, with the three TSPO ligands. **B,D,F**: Relative iNOS protein expression levels normalized to β-actin in pre-treatment, co-treatment, and post-treatment, respectively, with TSPO ligands. Results were calculated using densitometry and presented as means ± SEM; *n* = 4 (pre-treatment), *n* = 5 (co-treatment), *n* = 3 (post-treatment). * *p* < 0.05, *** *p* < 0.001 compared to Control; ### *p* < 0.001 compared to LPS; @ *p* < 0.05, @@ *p* < 0.01, @@@ *p* < 0.001 compared to LPS + PK 11195.

BV-2 cells that were treated with 25 µM of TSPO ligands or vehicle (control) had no detectable iNOS protein expression (Figure 2A,B, ANOVA *p* < 0.0001). LPS alone increased iNOS protein expression levels. While one hour of pretreatment with 25 µM of PK 11195 before LPS treatment resulted in 44% decrease in iNOS levels (Bonferroni *p* < 0.001), pretreatment with 25 µM of 2-Cl-MGV-1 or with 25 µM of MGV-1 reduced iNOS levels by 70% and 82%, respectively, compared to iNOS levels in LPS-treated BV-2 cells (Bonferroni *p* < 0.001, for both). Post hoc analysis that compared the effects of pretreatment with the three ligands showed that iNOS levels in LPS + MGV-1-treated cells were significantly lower than COX-2 levels in LPS + PK 11195-treated cells (Bonferroni *p* < 0.01).

PK 11195 and 2-Cl-MGV-1 added to LPS still showed significantly higher iNOS levels than control untreated cells (Bonferroni *p* < 0.001 for LPS + PK 11195 vs. Control and *p* < 0.05 for LPS + 2-Cl-MGV-1 vs. Control). In contrast, expression levels of iNOS in LPS + MGV-1-treated cells did not differ significantly from iNOS levels in control untreated cells (Bonferroni *p* > 0.05).

#### 2.3.2. Cotreatment

As in the pretreatment scenario, 25 µM of TSPO ligands did not affect iNOS levels (Figure 2C,D, ANOVA *p* < 0.0001). Cotreatment of LPS with 25 μM PK 11195, 2-Cl-MGV-1, and MGV-1 reduced iNOS levels by 36%, 59%, and 73%, respectively, compared to LPS-treated cells (Bonferroni *p* < 0.001, for each comparison). The effect of cotreatment with MGV-1 on iNOS levels was significantly larger than cotreatment with PK 11195 effect (Bonferroni *p* < 0.001). However, cotreatment with the three ligands was not able to reduce iNOS levels to the levels in control untreated cells (Bonferroni *p* < 0.001 for LPS + PK 11195 vs. Control, *p* < 0.001 for LPS + 2-Cl-MGV-1 vs. Control, *p* < 0.05 for LPS + MGV-1 vs. Control).

#### 2.3.3. Post-Treatment

As with the former scenarios, TSPO ligands (25 µM) without the presence of LPS had no effect by themselves on iNOS levels in BV-2 cells (Figure 2E,F, ANOVA *p* < 0.0001). Post-treatment with PK 11195, 2-Cl-MGV-1, and MGV-1 (25 µM) following one hour of exposure to LPS resulted in 53%, 57%, and 80% decrease in iNOS levels, respectively, compared to iNOS levels in LPS-treated BV-2 cells (Bonferroni *p* < 0.001, for each comparison). Post hoc analysis for comparison of iNOS levels between LPS + PK 11195-, LPS + 2-Cl-MGV-1-, and LPS + MGV-1-treated cells showed that only the MGV-1 effect on iNOS levels was significantly larger than the PK 11195 effect (Bonferroni *p* < 0.05). Also, among the three ligands, only post-treatment with MGV-1 reduced the iNOS expression to a level that was not statistically different from iNOS levels in control untreated cells (Bonferroni *p* > 0.05). In contrast, PK 11195 and 2-Cl-MGV-1 added one hour after LPS showed significantly higher iNOS levels than control cells (Bonferroni *p* < 0.001 for LPS + PK 11195 vs. Control and Bonferroni *p* < 0.001 for LPS + 2-Cl-MGV-1 vs. Control).

### 2.4. Effect of TSPO Ligands on Nitric Oxide (NO) Levels in LPS-Treated BV-2 Cells

#### 2.4.1. Pretreatment

**Figure 3:**
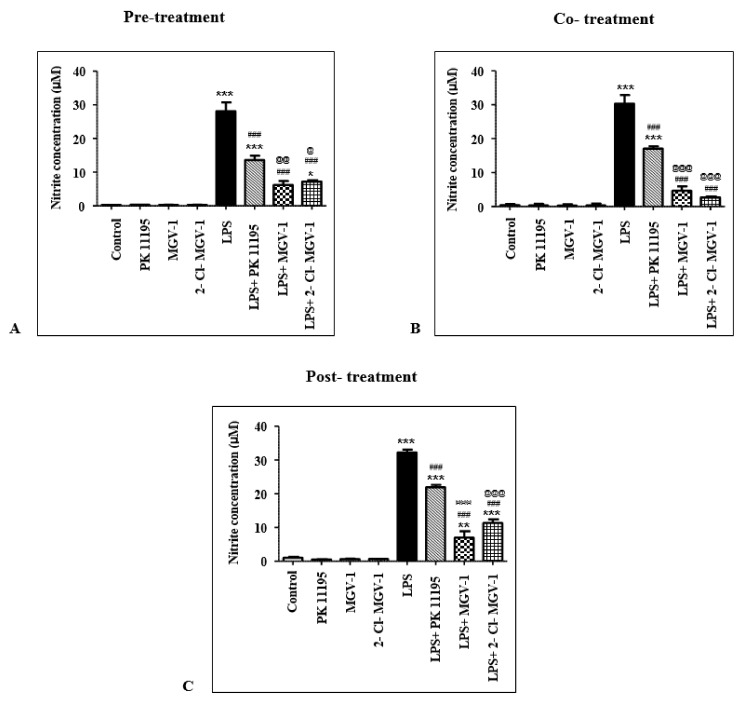
TSPO ligands reduced nitrite levels in LPS-treated BV-2 cells. BV-2 cells were treated with 100 ng/mL LPS for 24 h, with or without TSPO ligands (25 μM) in three different scenarios. **A**,**B,C**: Nitrite concentrations (μM) in pretreatment, cotreatment, and post-treatment, respectively, with TSPO ligands. Nitrite levels are presented as means ± SEM; *n* = 3 (pretreatment), *n* = 3 (cotreatment), *n* = 3 (post-treatment). * *p* < 0.05, ** *p* < 0.01, *** *p* < 0.001 compared to Control; ### *p* < 0.001 compared to LPS; @ *p* < 0.05, @@ *p* < 0.01, @@@ *p* < 0.001 compared to LPS + PK 11195.

NO levels in TSPO-ligand-treated BV-2 cells were not different from untreated control BV-2 cells (Figure 3A, ANOVA *p* < 0.0001). Compared to control untreated cells, LPS caused a 97-fold increase in NO levels (Bonferroni *p* < 0.001).

Although pretreatment with 25 µM of the TSPO ligands in LPS-treated BV-2 cells considerably diminished NO levels, pretreatment with PK 11195 reduced NO levels by 51% while 2-Cl-MGV-1 and MGV-1 reduced NO levels by 78% and 74%, respectively, compared to LPS-treated BV-2 cells (Bonferroni *p* < 0.001, for both). The effects of pretreatment with 2-Cl-MGV-1 or MGV-1 on NO levels were statistically greater than PK 11195 effect (Bonferroni *p* < 0.01, for LPS + 2-Cl-MGV-1 vs LPS + PK 11195, Bonferroni *p* < 0.05 for LPS + MGV-1 vs LPS + PK 11195).

NO levels in LPS + 2-Cl-MGV-1-treated cells were not significantly different from NO levels in control untreated cells (Bonferroni *p* > 0.05). In contrast, NO levels in both LPS + PK 11195- and LPS + MGV-1-treated BV-2 cells were higher than NO levels in control BV-2 cells (Bonferroni *p* < 0.001 for LPS + PK 11195 vs. Control and Bonferroni *p* < 0.05 for LPS + MGV-1 vs. Control).

#### 2.4.2. Cotreatment

Similar to the former scenario, NO levels in TSPO-ligand-treated BV-2 cells were similar to NO levels in control untreated BV-2 cells (Figure 3B, ANOVA *p* < 0.0001). Cotreatment of LPS with 25 µM of PK 11195, 2-Cl-MGV-1, and MGV-1 reduced NO levels by 44%, 85%, and 91%, respectively, compared to LPS-treated cells (Bonferroni *p* < 0.001, for both). However, the effects of cotreatment with 2-Cl-MGV-1 or MGV-1 on NO levels were significantly larger compared to the effect of cotreatment with PK 11195 (Bonferroni *p* < 0.001, for both).

NO levels in both LPS + 2-Cl-MGV-1- and LPS + MGV-1-treated BV-2 cells were not different from NO levels in control BV-2 cells (Bonferroni *p* > 0.05). In contrast, NO levels in LPS + PK 11195-treated BV-2 cells were larger than NO levels in control BV-2 cells (Bonferroni *p* < 0.001).

#### 2.4.3. Post-Treatment

As with the former scenarios, TSPO ligands (25 µM) had no effect on NO levels by themselves (Figure 3C, ANOVA *p* < 0.0001). Treatment of BV-2 cells with TSPO ligands starting one hour after LPS treatment significantly reduced NO levels compared to LPS-treated cells. While NO levels in LPS + PK 11195-treated cells decreased by 32% (Bonferroni *p* < 0.001), NO levels in LPS + 2-Cl-MGV-1- and LPS + MGV-1-treated cells decreased by 78% (Bonferroni *p* < 0.01) and 65%, respectively, compared to NO levels in LPS-treated BV-2 cells (Bonferroni *p* < 0.001). Post hoc analysis for comparison of the effects of post-treatment with each of the ligands on NO levels showed that PK 11195 was significantly less effective in reducing NO levels (Bonferroni *p* < 0.001 for LPS + PK11195 vs LPS + 2-Cl-MGV-1 or vs LPS + MGV-1).

However, NO levels following treatment with the three ligands remained significantly higher compared to control cells (Bonferroni *p* < 0.001 for LPS + PK 11195 vs. Control, Bonferroni *p* < 0.01 for LPS + 2-Cl-MGV-1 vs. Control, Bonferroni *p* < 0.001 for LPS + MGV-1 vs. Control).

## 3. Discussion

The current study aimed to examine whether the TSPO ligands 2-Cl-MGV-1 and MGV-1 are more efficient in mitigating neuroinflammatory response to LPS in comparison to the classic ligand, PK 11195. As expected, LPS treatment resulted in a significant increase in COX-2, iNOS, and NO levels, compared to control untreated BV-2 cells. COX-2, iNOS, and NO are produced by activated microglia [1]. COX-2 synthesizes prostaglandins, which are small lipid molecules that mediate an inflammatory response via their binding and activation of specific G-protein-coupled receptors (GPCR) [46]. NO functions as a neurotransmitter and partakes in signaling cascades that operate in a pathway between cerebral blood vessels, neurons, and glial cells. It is synthesized from arginine by the enzyme iNOS [47].

All three TSPO ligands were able to reduce the elevations in COX-2, iNOS, and NO levels, either when applied simultaneously with LPS for 24 h, 1 hour before LPS exposure, or 1 hour following LPS exposure. Thus, it appears that these ligands can reduce the inflammatory responses induced by LPS.

Our data support previous studies in which TSPO ligands attenuated neuroinflammation and neuropathology both in vitro and in vivo [42,44,48,49,50]. For example, Bonsack et al. [42] has shown that the TSPO ligand Ro5-4864 reduced the release of the proinflammatory cytokines IL-6 and TNF-α following microglial cells’ exposure to hemin. In another study, Ro5-4864 attenuated hippocampal β-amyloid accumulation and reduced gliosis in aged 3xTgAD mice that serve as a model of Alzheimer’s disease [48].

However, the mechanism by which TSPO regulates the inflammatory pathways in activated microglial cells is still not clear. One explanation is that TSPO ligands induce the production of neurosteroids that exert anti-inflammatory and neuroprotective effects [51,52,53]. This may explain why microglial cells, once activated, increase their TSPO expression; the increased TSPO levels may present a protective mechanism geared to attenuate the induced inflammation. Indeed, Santoro et al. [54] reported that TSPO ligands protected C6 glioma cells from LPS/IFNγ-induced inflammation in association with neurosteroid (pregnenolone) synthesis.

A second explanation is related to the ability of TSPO to modulate the nuclear factor-kappa B (NF-κB) pathway. It was shown that TSPO overexpression in a microglial cell line [41] or treatment with TSPO ligands [41,44] attenuated the activation of NF-κB, which regulates the transcription of various proinflammatory molecules. Thus, it is possible that TSPO modulates the inflammatory response of microglial cells, at least partly, through the NF-κB signaling pathway.

Third, TSPO ligands or TSPO overexpression in microglial cells increased the expression levels of M2-microglia-related genes [41]. M2 microglia are responsible for tissue repair and extracellular matrix (ECM) reconstruction. They also contribute to neuronal survival by enhancing neurotrophic factors, such as insulin-like growth factor-1 (IGF-I) [55]. Thus, it is possible that TSPO is involved in microglial transition from a “classical” activated phase to a more immunosuppressive and repair phase. Further research is needed to clarify the mechanisms that are involved in the anti-inflammatory activity of the new TSPO ligands. It is possible that the effect on the immune system is achieved by regulatory activity at the NF-κB pathway or an impact on the synthesis of neurosteroids.

In the present study, our new synthesized ligands, 2-Cl-MGV-1 and MGV-1, were found to be more efficient than PK 11195 in reduction of the tested inflammatory markers. A putative advantage of the new ligands is their relatively low affinity to the TSPO, potentially avoiding lethality otherwise caused by relatively high concentrations of high-affinity TSPO ligands [22,45].

Notably, 2-Cl-MGV-1 and MGV-1 were effective in our glial model even when administered one hour after the exposure to LPS, meaning that TSPO ligands possibly affect the events that occur immediately after induction of inflammation by LPS. This assumption is supported by previous studies in which TSPO was shown to regulate the expression of coding and noncoding genes in U118MG glioblastoma cells [19,56]. It is possible that TSPO can modulate the expression of genes that regulate the inflammatory response cascade also in microglial cells. 

Our cell culture in vitro results do not indicate major differences between the two new ligands, 2-Cl-MGV-1 and MGV-1, regarding potency in modulation of the inflammatory processes in microglial cells. One in vivo animal study conducted in our laboratory has shown that 2-Cl-MGV-1 can increase the lifespan of Huntington disease model mice, while MGV-1 was devoid of such an effect [22]. Further research is needed in order to test which of the two new ligands is more effective in diminishing neuroinflammation.

## 4. Materials and Methods

### 4.1. Cell Culture

Our in vitro model was BV-2 cells (generously provided by Zvi Vogel’s laboratory, Weizmann Institute of Science, Rehovot, Israel), derived from raf/myc-immortalized murine neonatal microglia. BV-2 microglia cells were cultured in Dulbecco’s modified Eagle’s medium (DMEM) containing 4.5 g/L glucose and 1 mM sodium pyruvate, supplemented with 5% fetal bovine serum, penicillin (100 U/mL), and streptomycin (100 μg/mL) [41]. Cells were incubated at 37°C, 5% CO_2_, and 90% relative humidity. 

### 4.2. Ligand Synthesis

The TSPO ligands were produced according to the state-of-the-art methods. All reactions were performed under argon atmosphere in flame-dried glassware and were monitored using analytical thin-layer chromatography (TLC). Absorbance at 254 nm wavelength, with silica-gel-coated glass plates with F254 indicator, was monitored. Then ^1^H and ^13^NMR were recorded on different spectrometers, and chemical shifts were reported. Atmospheric Pressure Photoionization Source (APPI) was used to achieve high-resolution mass spectrum (HRMS). Also, column chromatography and production of 2-arylquinazolin-4-ol were performed. The reaction mixture was further manipulated as described previously, and the resultant product was purified using silica gel chromatography and finally recrystallized using dichloromethane (DCM)/ethylacetate/n-pentane [22].

### 4.3. Procedures

In order to induce inflammatory responses in our model, we treated BV-2 cells with a final concentration of 100 ng/mL of LPS for 24 h. These cells are the most frequently used model for primary microglia studies (including pharmacological and immunological investigations). Notably, BV-2 cells treated with LPS had a response pattern similar to that of primary microglia [57].

The effects of 2-Cl-MGV-1 and MGV-1 on inflammatory response of BV-2 cells were compared to the classical TSPO ligand PK 11195. The three TSPO ligands were added at a fixed concentration of 25 µM in three different scenarios: pretreatment, co-treatment, and post-treatment. Each treatment scenario lasted for a total of 24 h. In each scenario, the experiments included the following four groups: Vehicle control group—cells that were treated with 1% vehicle-containing medium; LPS group—cells that were treated with a final concentration of 100 ng/mL of LPS, with vehicle but without ligand application; Ligand group—cells that were incubated with each of the three TSPO ligands without LPS; and LPS + Ligand group—cells that were incubated with each of the three TSPO ligands along with 100 ng/mL of LPS).

After 24 h, we measured the levels of three inflammatory markers, COX-2, iNOS, and NO, and compared the effects of the two novel TSPO ligands, MGV-and 2-Cl-MGV-1, to the effect of the classic TSPO ligand PK 11195.

### 4.4. Lipopolysaccharide (LPS) Treatment

BV-2 cells were seeded into 100 mm plates. After 48 h, cells were treated with 100 ng/mL lipopolysaccharide (LPS) (Sigma-Aldrich, Rehovot, Israel) for 24 h, as previously described [41]. Following LPS treatment, the cells were collected for the different assays described below.

### 4.5. TSPO Ligand Treatments

BV-2 cells were treated with a final concentration of 100 ng/mL of LPS with or without 25 µM of the TSPO ligands PK 11195, 2-Cl-MGV-1, and MGV-1. This concentration of the TSPO ligands was chosen since it was found, after several dose–response assays in our laboratory, to be effective in cell culture experiments [22,58]. The ligands were added in three different scenarios: pretreatment starting one hour before LPS treatment, co-treatment simultaneously with LPS, and post-treatment (LPS first and one hour later addition of ligands). Control cells were treated with vehicle (1% ethanol).

### 4.6. Western Blot

Following 24 h of the different treatments, cells were collected and prepared for Western blot analysis as described previously [59]. Briefly, cells were trypsinized, collected together with their culture medium, and centrifuged (210× *g* for 5 min at 4 °C). Cell pellets were lysed with lysis buffer (pH 7.4) containing 1% Triton X 100, 100 µL of protease inhibitors cocktail (Sigma-Aldrich, Rehovot, Israel) per 10 mL PBS, and 0.1% SDS dissolved in PBS. Total protein was measured according to Bradford (1976) [60] using BSA as a standard. Equal quantities of total protein (20 µg) were electrophoresed in SDS polyacrylamide gels (8% or 12%), transferred to a nitrocellulose membrane, and blocked with 4% BSA in Tris-buffered saline Tween (TBS-T). Then, the membranes were immunoblotted overnight at 4°C with primary antibodies including rabbit anti-mouse COX-2 (1:1000) (Abcam, Cambridge, UK) and rabbit anti-mouse iNOS (1:4000) (Abcam, Cambridge, UK). Mouse anti-mouse β-actin (1:15,000) (Santa Cruz Biotechnology, Dallas, TX, USA) was used as a loading reference. After washing with TBS-T, the membranes were incubated with IgG secondary antibody linked to horseradish peroxidase (anti-rabbit IgG 1:20,000, anti-mouse IgG 1:10,000) (Jackson Immunologicals, West Grove, PA, USA) in TBS-T for 1 hr at room temperature.

Binding of antibodies to the membrane was detected using EZ-ECL-detection reagent (Biological Industries, Beit Haemek, Israel) with either X-Omat blue XB-1 Kodak Scientific Imaging Films or with ImageQuant LAS4010 imager (GE Healthcare, Life Science, Pittsburgh, PA, USA). The results were analyzed by densitometry, with TotalLab Quant software (TotalLab Ltd., Newcastle, UK).

### 4.7. Nitrite Assay

Nitrite (NO_2_^−1^), which is one of the final products in nitric oxide (NO) oxidation, serves as a marker for NO production and its levels are correlated with NO levels [61]. NO production was determined by measuring the levels of nitrite in the cells’ supernatant using a colorimetric reaction with Griess reagent (Sigma, Rehovot, Israel) according to the manufacturer’s instructions. Briefly, cells were seeded in 10 mm culture plates, and 48 h later were treated with LPS and TSPO ligands according to the above protocol. After 24 h of treatment, equal volumes of cell supernatants and Griess reagent were mixed. Sodium nitrate was used to build a calibration curve. Absorbance was measured at 540 nm after 15 mins, with Spectrophotometer Zenyth 200 (Anthos, Eugendorf, Austria).

### 4.8. Statistical Analysis

Results are presented as Mean ± Standard Error of Mean (SEM). One-way analysis of variance (ANOVA) test was used as appropriate, with Bonferroni correction as a post hoc test. Statistical significance was defined by *p* < 0.05. Data were analyzed by GraphPad Prism 7- for Windows (GraphPad Software, La Jolla, CA, USA).

## 5. Conclusions

In the present study, we have shown that our new synthesized ligands, 2-Cl-MGV-1 and MGV-1, are more efficient than PK 11195 in reduction of inflammatory markers such as COX-2, iNOS, and NO in glial cells. These findings indicate that our novel TSPO ligands, 2-Cl-MGV-1 and MGV-1, may serve as potential therapeutics in neuroinflammatory and neurodegenerative diseases.
